# Thyroid-like low-grade nasopharyngeal papillary adenocarcinoma: A case report

**DOI:** 10.1097/MD.0000000000021599

**Published:** 2020-08-14

**Authors:** Li Maocai, Liu Fuxing, Li Lianqing, Gong Lili, Duan WenChao, Liu ShouZhou

**Affiliations:** Department of Otolaryngology, Head and Neck Surgery, Liaocheng People's Hospital, Liaocheng, Shandong, China.

**Keywords:** adenocarcinoma, nasopharynx, thyroid transcription factor-1, fluorodeoxyglucose-positron emission tomography-computed tomography, magnetic resonance imaging

## Abstract

**Introduction:**

Thyroid-like low-grade nasopharyngeal papillary adenocarcinoma (TL-LGNPPA) is a rare neoplasm characterized by morphological analogy to papillary thyroid carcinoma and the abnormal expression of thyroid transcription factor-1 (TTF-1). We herein report a rare case of TL-LGNPPA with a review of its clinical and morphological characteristics and the treatment provided.

**Patient concerns and Diagnosis:**

The patient was a 50-year-old Chinese woman with the complaint of a three-year history of phlegm with blood with pharyngeal discomfort. There were no remarkable physical findings, and the laboratory tests were normal. Laryngoscopy and nasal computed tomography identified a mass at the posterior end of the nasal septum. Histologically, the tumor exhibited an oval papillary growth. Immunohistochemically, the neoplastic cells were positive for TTF-1, vimentin, cytokeratin 7, and cytokeratin 19. Pathological examination indicated a thyroid-like low-grade nasopharyngeal papillary adenocarcinoma.

**Intervention:**

The neoplasm was completely resected without any complication.

**Outcomes:**

The patient had neither local recurrence nor distant metastasis 1 year after the removal of the tumor.

**Conclusion:**

Although TL-LGNPPA is a malignant tumor, complete surgical resection is an effective treatment.

## 1 Introduction

Primary nasopharyngeal adenocarcinoma is particularly rare, comprising <0.5% of the cases of malignant nasopharyngeal neoplasms. Low-grade nasopharyngeal papillary adenocarcinoma (LGNPPA) was first described and characterized by Wenig et al in 1988, and since then the number of published case reports has been limited (1). Thyroid-like LGNPPAs (TL-LGNPPAs) represent a minority of LGNPPAs, and they are characterized by the abnormal expression of thyroid transcription factor-1 (TTF-1). To the best of our knowledge, only 13 cases have been reported in the English literature, and 12 have been reported in the Chinese literature to date. In this article, we presented the case of primary TL-LGNPPA and an analysis of its treatment experience.

## 2 Case report

**Figure 1:**
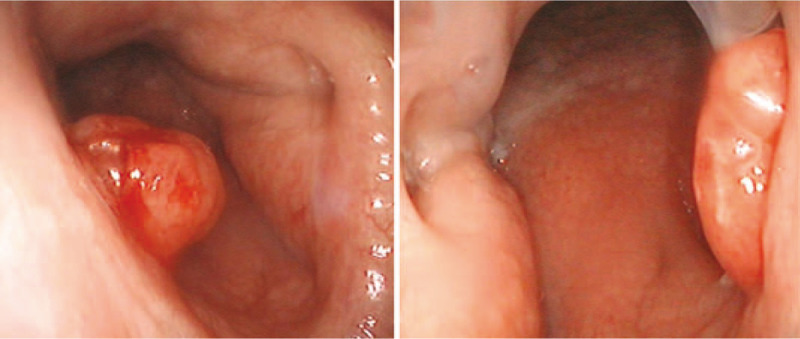
A red, peanut-sized neoplasm with a smooth surface, soft texture, and hemorrhagic tendency on the free margin of the caudal nasal septum. It can be seen from both sides of the bilateral nasal cavity, and the nasopharyngeal mucosa on both sides is smooth.

**Figure 2:**
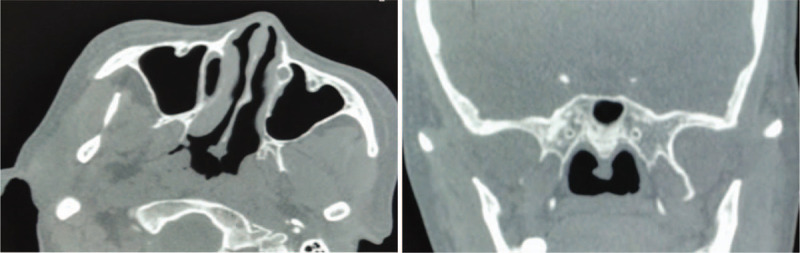
Computed tomography showing a soft tissue shadow on the caudal nasal septum.

A 50-year-old woman visited our hospital on March 27, 2016, with chief complaints of bloody phlegm accompanied by pharyngeal sensation of a foreign body for 3 years. The patient had no history of hoarseness, chest tightness, dyspnea, dysphagia, nasal obstruction, headache, dizziness, or tinnitus. Before the visit, the patient had visited a local hospital where the patient was diagnosed with gingival bleeding and pharyngitis and treated using antibiotics. However, the treatment was not effective. At our hospital, laryngoscopy identified a red, peanut-sized neoplasm with a smooth surface, soft texture, and hemorrhagic tendency on the free margin of the caudal nasal septum. Nasopharyngeal mucosa on both sides was smooth (Fig. 1). Considering the possibility of hemangioma, we admitted the patient for surgery. Physical examination performed after admission revealed no enlarged lymph nodes in the head and neck regions. However, axial computed tomography (CT) revealed a round and soft tissue shadow on the caudal nasal septum (Fig. 2). Preoperative tests were comprehensively conducted, and no obvious contraindication of surgery was found. Accordingly, endoscopic resection of the nasopharyngeal mass was performed under general anesthesia. During resection, the bilateral nasal mucosa was contracted, and no obstruction of the bilateral nasal cavity was detected. Meanwhile, a red neoplasm with a smooth surface and hemorrhagic tendency was detected in the caudal nasal septum. The pedicle flap of the neoplasm was located in the upper third portion of the right side of the caudal nasal septum, and the mucosa around the pedicle flap appeared normal. Mucosal resection was performed approximately 0.5 cm around the root of the pedicle flap. Subsequently, the neoplasm was completely resected. The 1.2 × 0.8 × 0.4 cm resected neoplasm was checked to ensure its integrity. After marking, the sample was sent for pathological examination. Erythromycin ointment was applied to the wound following hemostasis by electrocoagulation. The patient provided written informed consent for the publication of the case report.

Postoperatively, an anti-inflammatory treatment was administered. Notably, no complications were detected. Pathological examination suggested a thyroid-like low-grade nasopharyngeal papillary adenocarcinoma. Immunohistochemical study indicated the following: CK (+), vimentin (+), TTF-1 (+), CK19 (+), Ki67 (1%+), TG (−), CK5/6 (−), and CD117 (−); these results confirmed the above diagnosis. Nasal endoscopy was repeated after postoperative 7 days, and the recovery of the incision site was found to be good. The patient was accordingly discharged, and no recurrence was reported during 2-year follow-up.

## 3 Discussion

Thyroid-like low-grade papillary adenocarcinoma was first reported by Wenig in 1998.^[1]^ This tumor is histopathologically a type of rarely reported epithelial- or non-epithelial-originating tumor characterized by various histological morphologies and biological behaviors. Further, thyroid-like low-grade nasopharyngeal papillary adenocarcinoma was first reported and named by Carrizo.^[2]^ Its clinical diagnosis involves the combined use of histological examination and identification of immunophenotypes characterized as being strongly positive for TTF-1, CK7, and CK19.

According to the literature, thyroid-like low-grade nasopharyngeal papillary adenocarcinomas can occur at any age and presents no sex-specific difference in terms of incidence. The primary symptoms include nasal obstruction, epistaxis, and other related symptoms such as tinnitus, ear tightness, hearing loss, and neurological disorders.^[3,4]^ We believe that the symptoms are associated with the size, location, and extent of the tumor. The main manifestation of this case was bloody sputum with pharyngeal sensation of a foreign body. The tumor can be exogenous, papillary, nodular, or lobulated on nasopharyngoscopy with a soft texture. In the present case, CT showed a low-density shadow in the soft tissue. The tumor can occur in any part of the nasopharynx. According to relevant reports, it is commonly observed on the top, lateral, and posterior walls of the nasopharynx as well as on the free margin of the caudal nasal septum. In this case, the neoplasm was located on the free margin of the caudal nasal septum. Based on the clinical symptoms and characteristics mentioned, the tumor was initially diagnosed to be benign. The cause of this type of tumor remains unknown. However, it is not related to EBV and HPV infection.^[5,6]^

Excision is considered the first-line treatment for thyroid-like low-grade nasopharyngeal papillary adenocarcinoma. Complete excision based on the size, location, and extent of the tumor can obtain desirable effects. According to national and international reports on this type of tumor, remarkable clinical outcomes have been observed following its surgical resection. To date, no cases of recurrence have been reported with the follow-up period of as long as 15 years.^[2]^

As a rare malignant neoplasm of the nasopharynx, thyroid-like low-grade nasopharyngeal papillary adenocarcinoma is preoperatively often misdiagnosed as a benign tumor. Its diagnosis depends on postoperative histological and immunohistochemical examination. Therefore, attention should be paid to this type of tumor in clinical practice and its possibility should be considered when dealing with similar cases. Preliminary diagnosis is associated with the choice of surgery, extent of resection, and whether the tumor should be completely resected. Complete resection of the tumor determines the clinical effect.

## Author contributions

Guarantor of integrity of entire study: Li Lianqing Study concepts: Li Lianqing Study design: Gong Lili Literature research: Gong Lili Clinical studies: Li Maocai Data acquisition: Liu Shouzhou Data analysis/interpretation: Liu Fuxing Statistical analysis: Liu Fuxing Manuscript preparation: Li Maocai.

Manuscript defnition of intellectual content: Duan Wenchao Manuscript editing: Duan Wenchao Manuscript revision/review: Li Maocai Manuscript final version approval: Li Lianqing.
